# Left-sided thoracic curves in adolescent idiopathic scoliosis: are the outcomes different?

**DOI:** 10.1007/s43390-025-01069-0

**Published:** 2025-04-02

**Authors:** Bryan O. Ren, Anne Marie Dumaine, Jerry Y. Du, Daniel J. Hedequist, Michael P. Glotzbecker, Harms Study Group

**Affiliations:** 1 Department of Orthopaedic Surgery, University of Michigan, Ann Arbor, MI USA; 2 Shriners Children’s Lexington, Lexington, KY USA; 3 Virginia Mason Franciscan Health, Seattle, WA USA; 4 Department of Orthopedic Surgery, Boston Children’s Hospital, Boston, MA USA; 5 University Hospitals Rainbow Babies and Children’s Hospital Division of Pediatric Orthopedics, 11100 Euclid Avenue, Cleveland, OH 44106 USA

**Keywords:** Adolescent idiopathic scoliosis, Left-sided thoracic, Left-sided curve

## Abstract

**Purpose:**

The purpose was to compare outcomes in adolescent idiopathic scoliosis (AIS) patients with left vs. right main thoracic curves. A secondary purpose was to elucidate surgeon opinions between these two curve types and differences in surgical approach via a survey.

**Methods:**

A multicenter database was utilized to identify AIS patients with left thoracic curves without significant MRI abnormalities from 1996 to 2018 receiving posterior spinal fusion. A 1:1 propensity match was performed to right thoracic curves. Differences between the curve groups were analyzed.

**Results:**

Fifty patients had left thoracic curves. Curve direction had no impact on levels fused, surgical time, length of hospitalization, estimated blood loss, cell saver transfused, death, pain, pseudoarthrosis, reoperation, or infection rates. At two-years postoperatively, left curves had greater T2-T12 kyphosis (37 vs. 31°, p = .02), and variations in T1 tilt angle direction (30 vs. 39 left tilt, p = .04), EIV angulation (-2 vs. 5°, p < .01), EIV translation (0.6 vs. -0.2 cm, p = .03), and left shoulder elevation (12 in left curves vs. 25 in right curves, p = .01). There were no differences in correction rate, Cobb angle, lumbar curve, T12-S1 lordosis, shoulder height difference, or SRS 22 scores.

**Conclusion:**

In AIS, there are no significant differences in surgical characteristics or outcomes between left and right-thoracic curves. Notably, curve direction does not influence correction rate, surgical time, levels fused, length of hospitalization, blood loss, pain, SRS 22 scores, or rates of reoperation, infection, or pseudoarthrosis. There are some variations in radiographic parameters postoperatively that are unlikely to be clinically significant.

## Introduction

The majority, an estimated 90%, of patients with a main thoracic curve in adolescent idiopathic scoliosis (AIS) will have the convexity directed toward the right [1, 2]. Left thoracic curves are rarer and may be associated with smaller and less progressive curves [3]. However, when patients present with left-sided main thoracic curves, additional workup is necessary to examine for other etiologies. An MRI is typically conducted due to concern for intraspinal pathology [4–6].

Even when intraspinal and other etiologies have been ruled out, patients with left-sided thoracic curves present challenges in the operating room. Many surgeons will typically stand on the left-hand side of the patient, and therefore instrument the concave pedicles. The change in directionality may influence the normal routine of the surgeon which could negatively impact outcomes. Things that may be considered determine the side the attending surgeon stands on, the side the first assistant places screws, and which rod is placed first. There are also anatomic features that need to be considered, such as the position of the aorta regarding implant placement and deformity correction [7–9].

There are limited literatures comparing surgical outcomes in left versus right main thoracic curves. One study found similar radiographic and functional outcomes between left and right major thoracic curves treated surgically, with a nonsignificant longer operative time and more estimated blood loss in left-sided thoracic curves [10]. However, this study was conducted at a single institution and limited to 12 patients. To the authors’ best knowledge, there have not been any large multicenter studies analyzing the influence of curve direction on surgical outcomes in AIS. Therefore, the purpose of the present study was to compare perioperative and postoperative outcomes in AIS patients with left versus right-sided main thoracic curves. A secondary purpose was to describe how surgeons typically approach these curves through a survey. The authors hypothesized that left-sided thoracic curves in AIS patients require longer operative time and greater blood loss, and suffer from more complications than similar curves with a thoracic apex to the right.

## Materials and methods

**Fig. 1:**
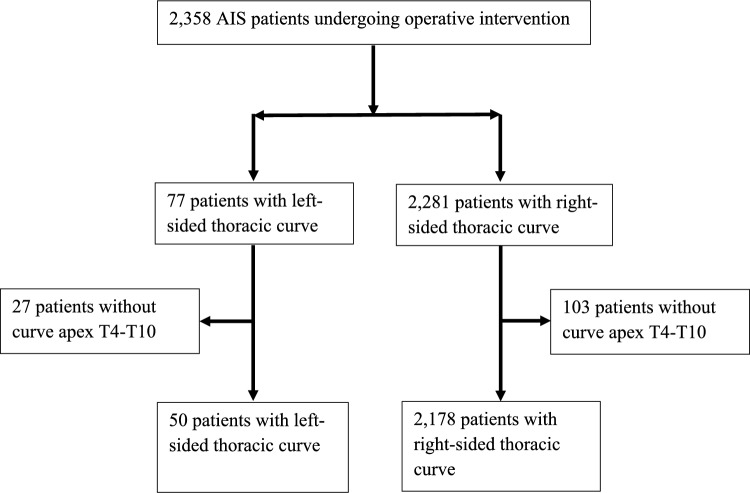
The CONSORT flowchart of the study

A multicenter database from an international study group was used to identify patients with AIS from 1996 to 2018. The study population was isolated to patients with thoracic curves having an apex in the range of T4–T10. We excluded those with an apex at T11 and T12 as it has been demonstrated these curves may behave differently than typical thoracic curves [11]. Our final cohort consisted of 50 patients with left-sided and 2,178 patients with right-sided thoracic curves (Fig. 1).

A 1:1 propensity score match was performed between the two groups using the variables of age at index surgery, sex, race, location of surgery site, and curve magnitude. Chi-squared, Fisher’s exact test, and independent samples *t*-tests were conducted to analyze differences between the groups. All statistical analyses were performed with the Statistical Package for the Social Sciences software for Mac version 28.0 (IBM Corp, Armonk, New York), with an alpha value of < 0.05 to determine statistical significance. A survey was also sent out to members of a nationally respected study group to better characterize individual surgeon preferences, experiences, and opinions when operating on left-sided curves.

## Results

A total of 50 patients with left-sided main thoracic curves were matched to 50 patients with right-sided thoracic curves. Preoperatively, left-sided curve patients had differences in T1 tilt angle (9.3° vs. 4.4°, *p* < 0.001), direction of EIV angulation (−9.2° vs. 15.2°, *p* < 0.001), thoracic kyphosis (37.1° vs. 27.2°, *p* < 0.001), SRS-22 mental health scores (4.0 vs. 4.2, *p* = 0.039), and greater proportion of the left shoulder being higher (22 vs. 8, *p* = 0.003) (Table 1).

**Table 1:** Pre-operative characteristics

	Left	Right	P-value
Height (cm)	163.9 (± 10.8)	164.8 (± 9.2)	0.661
Weight (kg)	56.9 (± 17.0)	57.5 (± 11.7)	0.862
Lumbar curve (degrees)	35.2 (± 11.3)	36.4 (± 11.2)	0.589
Thoracic bend (degrees)	33.9 (± 13.8)	36.4 (± 15.8)	0.41
Lumbar bend (degrees)	14.2 (± 10.8)	13.5 (± 10.6)	0.73
T1 tilt angle (degrees)	9.3 (± 6.6)	4.4 (± 3.9)	** < 0.001**
T1 tilt angle direction			
Blank	2	5	0.401
Right	16	18	
Left	32	27	
EIV angulation (degrees)	−9.2 (± 17.5)	15.2 (± 11.7)	** < 0.001**
EIV translation (cm)	0.3 (± 1.7)	−0.1 (± 1.6)	0.326
Risser stage			
Blank	1	2	0.19
0	6	3	
1	5	1	
2	1	6	
3	8	11	
4	23	19	
5	6	8	
Tri-radiate status			
Blank	1	0	0.45
Open	5	3	
Closed	44	47	
Kyphosis from T2-T12 (degrees)	37.1 (± 13.6)	27.2 (± 16.1)	** < 0.001**
Lordosis from Top T12—Top sacrum (degrees)	61.7 (± 14.4)	57.1 (± 10.7)	0.073
Junctional kyphosis proximal (degrees)	3.7 (± 5.9)	2.5 (± 4.8)	0.303
Junctional kyphosis distal (degrees)	−11.1 (± 10.4)	−8.3 (± 10.1)	0.183
Pelvic Incidence (degrees)	54.1 (± 14.4)	56.6 (± 11.4)	0.483
SRS-22 normalized scores			
Pain	4.0 (± 0.7)	4.2 (± 0.6)	0.19
Self-image	3.5 (± 0.7)	3.4 (± 0.7)	0.624
General function	4.4 (± 0.6)	4.5 (± 0.5)	0.457
Mental health	4.0 (± 0.6)	4.2 (± 0.6)	**0.039**
Satisfaction	3.5 (± 1.0)	3.6 (± 0.9)	0.964
Total	3.9 (± 0.5)	4.0 (± 0.4)	0.219
Shoulder height difference (cm)	1.3 (± 0.8)	1.0 (± 0.8)	0.211
Which shoulder high?			
Blank	21	21	**0.003**
Left	22	8	
Right	6	20	
Neutral	1	1	
Rib HumpT (degrees)	14.7 (± 5.6)	15.2 (± 4.9)	0.665
Rib HumpL (degrees)	6.7 (± 4.5)	6.7 (± 3.8)	0.953
Trunk shift (cm)	2.0 (± 1.4)	2.34(± 1.5)	0.264

Significant p values are in bold

At one year postoperatively, left-sided curves had variations in T1 tilt to the right (17 vs. 6, *p* = 0.016), thoracic kyphosis (35.0° vs. 29.3°, *p* = 0.007), lumbar lordosis (55.4° vs. 49.7°), EIV angulation (−1.3° vs. 3.6°, *p* < 0.001), and EIV translation (0.9° vs. −1.2°, *p* < 0.001) (Table 2). There were no differences between curve direction and correction rate, thoracic or lumbar curve degree, T1 tilt angle, proximal or distal junctional kyphosis, or pelvic incidence.

**Table 2:** 1-year postoperative outcomes

	Left	Right	P-value
Correction rate	0.7 (± 0.4)	0.7 (± 0.6)	0.922
Thoracic curve (degrees)	16.5 (± 7.8)	17.8 (± 5.1)	0.355
Lumbar curve (degrees)	13.9 (± 9.3)	15. (± 8.4)	0.538
T1 tilt angle (degrees)	5.3 (± 4.0)	5.5 (± 3.6)	0.835
T1 tilt angle direction			
Blank	4	2	**0.016**
Right	17	6	
Left	29	42	
EIV angulation (degrees)	−1.3 (± 6.7)	3.6 (± 5.3)	** < 0.001**
EIV translation (cm)	0.9 (± 1.3)	−1.2 (± 1.6)	** < 0.001**
Kyphosis from T2-T12 (degrees)	35.0 (± 9.5)	29.3 (± 10.9)	**0.007**
Lordosis from Top T12—top sacrum (degrees)	55.4 (± 14.3)	49.7 (± 10.6)	**0.028**
Junctional kyphosis proximal (degrees)	6.6 (± 4.5)	4.8 (± 4.7)	0.067
Junctional kyphosis distal (degrees)	−6.5 (± 9.5)	−4.9 (± 9.2)	0.394
Pelvic incidence (degrees)	51.8 (± 13.5)	55.2 (± 10.2)	0.328

Significant p values are in bold

At two years postoperatively, left-sided curves had differences in T1 tilt to the right (19 vs. 8, *p* = 0.036), thoracic kyphosis (36.5° vs. 30.8°, *p* = 0.017), proportion of left shoulder being higher (12 vs. 25, *p* = 0.005), EIV angulation (−2.2° vs. 4.5°, *p* < 0.001), and EIV translation (0.6° vs. −0.2°, *p* = 0.025) (Table 3). Similar to the one-year postoperative outcomes, curve direction did not influence correction rate, thoracic or lumbar curve, T1 tilt angle, proximal or distal junctional kyphosis, or pelvic incidence. Additionally, there was no difference in lordosis, shoulder height difference, rib hump, trunk shift, SRS-22 scores in each domain and total score, or any type of complication. Curve direction did not impact perioperative surgery characteristics or outcomes, in particular length of hospitalization, surgical time, blood loss (estimated blood loss and cell saver transfused), pain (day of conversion to oral pain medications and visual analog pain scale from immediate post operatively to postoperative day 4), number of levels fused, construct type, or use of de-rotation maneuvers (Table 4).

**Table 3:** 2-year postoperative outcomes

	Left	Right	P-value
Correction rate	0.6 (± 0.3)	0.6 (± 0.5)	
Thoracic curve (degrees)	19.1 (± 8.4)	20.2 (± 7.6)	0.526
Lumbar curve (degrees)	14.6 (± 8.9)	14.4 (± 9.0)	0.894
T1 tilt angle (degrees)	5.4 (± 4.7)	5.6 (± 3.9)	0.778
T1 tilt angle direction			
Blank	1	3	**0.036**
Right	19	8	
Left	30	39	
EIV angulation (degrees)	−2.2 (± 7.4)	4.5 (± 5.0)	** < 0.001**
EIV translation (cm)	0.6 (± 1.5)	−0.2 (± 1.7)	**0.025**
Kyphosis from T2-T12 (degrees)	36.5 (± 11.1)	30.8 (± 12.4)	**0.017**
Lordosis from Top T12—top sacrum (degrees)	60.4 (± 12.8)	56.1 (± 12.6)	0.099
Junctional kyphosis proximal (degrees)	6.7 (± 5.4)	5.4 (± 6.2)	0.251
Junctional kyphosis distal (degrees)	−7.5 (± 10.2)	−5.2 (± 11.3)	0.288
Pelvic Incidence (degrees)	57.7 (± 12.0)	52.3 (± 10.5)	0.097
SRS-22 normalized scores			
Pain	4.4 (± 0.6)	4.4 (± 0.7)	0.913
Self-Image	4.5 (± 0.5)	4.5 (± 0.6)	0.957
General function	4.5 (± 0.5)	4.5 (± 0.5)	0.826
Mental health	4.2 (± 0.7)	4.2 (± 0.8)	0.807
Satisfaction	4.6 (± 0.5)	4.6 (± 0.8)	0.854
Total	4.4 (± 0.4)	4.4 (± 0.5)	0.985
Shoulder height difference (cm)	0.9 (± 0.6)	1.2 (± 0.9)	0.065
Which shoulder high?			
Blank	21	18	**0.005**
Left	12	25	
Right	16	4	
Neutral	1	3	
Rib HumpT	7.4 (± 4.6)	7.0 (± 3.4)	0.639
Rib HumpL	4.4 (± 3.7)	3.2 (± 3.4)	0.155
Trunk shift	1.0 (± 0.8)	1.0 (± 0.9)	0.995
Complications			
Death	0	0	N/A
Gastrointestinal	1	2	1
Instrumentation	1	3	0.617
Medical	3	2	1
Neurologic	2	5	0.436
Pain	5	4	1
Pseudoarthrosis	1	0	1
Pulmonary	4	2	0.678
Reoperation	0	0	N/A
Surgical-site infection/incision	3	1	0.617
Transfusion	1	0	1

Significant p values are in bold

**Table 4:** Surgery characteristics

	Left	Right	P-value
Length of hospitalization (days)	5.2 (± 1.6)	5.7 (± 6.6)	0.582
Surgical time (min)	304.3 (± 111.4)	282.6 (± 117.6)	0.356
Antifibrinolytics—TXA	21	23	0.687
Antifibrinolytics—Amicar	2	3	0.646
EBL (cc)	985.7 (± 693.0)	1019.6 (± 935.6)	0.838
Cell saver transfused (cc)	268.1 (± 268.9)	348.4 (± 391.4)	0.271
Day conversion to only PO pain meds	3.1 (± 1.3)	2.8 (± 0.9)	0.238
Visual analog scale			
Post Op	6.0 (± 3.2)	6.2 (± 3.2)	0.75
POD 1	5.8 (± 2.3)	6.1 (± 2.7)	0.548
POD 2	6.2 (± 1.6)	5.8 (± 2.8)	0.447
POD 3	5.7 (± 1.9)	5.3 (± 2.3)	0.492
POD 4	5.0 (± 1.8)	4.8 (± 1.9)	0.749
Levels fused	11.3 (± 1.9)	11.2 (± 1.8)	0.701
Construct type			
Blank	1	1	0.927
80% screw	17	14	
All hook	1	1	
All screw	29	33	
Hybrid	2	1	
De-rotation maneuver performed?			
Blank	8	6	0.094
No	4	0	
Yes	38	44	

The results of the survey demonstrate variations in operative technique and opinions on left-sided thoracic curves (Table 5). Most surgeons stand on the same side in left- and right-sided thoracic curves (20/29, 69%) and place screws from the convex left side in left-sided curves (16/20, 80%). Understandably, surgeons noted giving fellows more autonomy with screw placement than residents, but this also depended on the time of the year and familiarity of the surgeon with the resident’s skill level. Most surgeons placed the concave rod first in right side (25/29, 86%) and left side (22/25, 88%) curves. Most surgeons did not feel that left-sided curves were more challenging (20/29, 69%), stiffer (21/29, 72%), associated with higher intraoperative blood loss (25/29, 86%), or associated with higher intraoperative neuromonitoring alerts (24/29, 83%) than right-sided curves. Overall, the consensus among the surgeons was that left-sided curves did not pose any significant challenges, but just had to adjust for the difference in sidedness.

**Table 5:** Survey responses

Typical first assistant?	Fellow	Resident	PA	Mixed	Other
	10/29 (34%)	10/29 (34%)	5/29 (17%)	2/29 (7%)	2/29 (7%)
Stand on same side in left-sided curves compared to right?	Yes	No	Depends on assistant		
	20/29 (69%)	7/29 (24%)	2/29 (7%)		
If stand on typical side (left) for left-sided curves, which side place screws?	Convex (left)	Concave (right)	Both	Depends on assistant	
	16/20 (80%)	1/20 (5%)	2/20 (10%)	1/20 (5%)	
If stand on right side for left-sided curves, which side place screws?	Convex (left)	Concave (right)	Both	Depends on assistant	
	0	3/7 (43%)	1/7 (14%)	3/7 (43%)	
Which rod usually placed first in right-sided curves?	Left (concave)	Right (convex)	Varies		
	25/29 (86%)	3/29 (10%)	1/29 (3%)		
If typically place left-sided rod (concave) first in right-sided curves, which rod placed first in left-sided curves?	Left (convex)	Right (concave)	Varies		
	1/25 (4%)	22/25 (88%)	2/25 (8%)		
If typically place right-sided rod (convex) first in ride sided curve, which rod placed first in left-sided curves?	Left (convex)	Right (concave)			
	2/3 (67%)	1/3 (33%)			
Feel that left-sided curves are more challenging than right?	Yes	No	Depends on assistant		
	7/29 (24%)	20/29 (69%)	2/29 (7%)		
Feel that left-sided curves are stiffer than right?	Yes	No			
	8/29 (28%)	21/29 (72%)			
Feel that left-sided curves are associated with higher intraoperative EBL than right?	Yes	No			
	4/29 (14%)	25/29 (86%)			
Feel that left-sided curves are associated with higher intraoperative neuromonitoring alerts than right?	Yes	No			
	5/29 (17%)	24/29 (83%)			

## Discussion

The present study examined perioperative and postoperative outcomes of AIS patients with left-sided compared to right-sided thoracic curves. At one and two years postoperatively, left-sided curves had variations in T1 tilt direction, thoracic kyphosis, EIV angulation and translation. Left-sided curves had more lumbar lordosis at one year but no difference at two years postoperatively. Right sided curves had a greater proportion of the left shoulder being higher at two years postoperatively. Beyond these variables, curve direction had no influence on perioperative and postoperative outcomes. At one and two years postoperatively, there was no difference in correction rate, thoracic or lumbar curve, T1 tilt angle, proximal or distal junctional kyphosis, or pelvic incidence. At two years postoperatively, there was no difference in lordosis, shoulder height difference, rib hump, trunk shift, SRS-22 scores in each domain and total score, or any type of complication including rates of reoperation, infection, or pseudoarthrosis. Curve direction did not impact perioperative surgery characteristics or outcomes, including length of hospitalization, surgical time, blood loss, pain, number of levels fused, construct type, or use of de-rotation maneuvers. Overall, it does not appear that there are clinically significant perioperative or postoperative differences between left- and right-sided thoracic curves in AIS, and the consensus among pediatric spine surgeons mirrors these results.

Our results are corroborated by a single institution study comparing 12 left-sided to 12 right-sided thoracic curve AIS patients, which found no difference in radiographic and or functional outcomes, including correction rate, complications, and SRS-22 scores [10]. However, the authors noted left-sided curves having a nonsignificant longer operative time, greater intraoperative estimated blood loss, and requiring greater volumes of blood transfusions. We found left-sided curves also had a nonsignificant increased operative time, but lower intraoperative estimated blood loss and cell saver transfusion requirements.

The left-sided curve cohort in our study had greater thoracic kyphosis both preoperatively and at two years postoperatively, which Wang et al. 2014 also found. Anecdotally, the authors of the present study also felt differences in kyphosis between left and right thoracic curves when operating. In the absence of intraspinal pathology, greater kyphosis in left-sided curves could be a co-existing phenotype [10]. Evidence supports anterior spinal overgrowth as a reason for hypokyphosis in patients with AIS [12]. Zhang et al. found those patients with hyperkyphosis also had a smaller pelvic incidence and sagittal vertical axis, theorizing that thoracic hyperkyphosis is a mechanism to achieve sagittal balance when the pelvis is unable to adjust appropriately [13]. Our data did not demonstrate differences in pelvic incidence between left and right curves. Although not the focus of the present investigation, future studies can be aimed at further exploring the relationship between curve direction, kyphosis, and pelvic parameters.

In terms of shoulder height at two years postoperatively, right-sided curves tended to have the left shoulder higher, whereas left-sided curves had similar distributions of left and right shoulder being higher. We are hesitant to make further hypotheses or conclusions on this result due to a proportion of missing data on this variable. There were differences in T1 tilt angle direction, and EIV angulation and translation. However, these radiographic differences are unlikely to be clinically significant.

Regardless, left-sided curves present challenges in the operating room from both a logistic and anatomic standpoint. In right-sided thoracic curves, the attending surgeon typically stands on the left (concave) side and places the concave rod first. There are differences in pedicle anatomy, and you must consider where a trainee may place screws in these cases. Left-sided curves present differences in the anatomy around the spine. The aorta is located more anterior and to the right (concavity) and the spinal cord is closer to the right-side pedicles [7, 8]. Therefore, care must be taken with right-sided pedicle screws, especially to ensure they are not too long. Left-sided curves have also been associated with dextrocardia, occasionally with complete situs inversus [14, 15].

When sidedness is flipped in left curves, our survey noted variation in the side the attending prefers to stand, and which rod is placed first. However, most surgeons stand on the same side in both left- and right-sided curves but will place the concave rod first. Most surgeons did not feel that left-sided curves were more challenging or associated with more intraoperative issues than right curves. Although, some surgeons noted being more cognizant of neurological changes and less familiar with left curves given their infrequency. It should be noted that this study included surgeons from high-volume centers, and the challenges of directionality may be a larger issue for those who encounter these cases less frequently.

There are several limitations to this study. Although the majority of the left-sided curve patients received an MRI with negative findings, a proportion of the patients either did not receive one (16) or MRI status was not documented (7). Two patients had findings on MRI, one was mild cerebellar tonsillar ectopia and the other was a small degenerative extraspinal synovial cyst involving the left L4-L5 facet joints. However, these did not preclude them from surgery and by design to be in this multicenter database, there cannot be significant MRI abnormalities. Given that this data is a compilation of numerous centers for a large time frame, some of the variables measured had incomplete data. The data are from tertiary referral hospitals which have relatively high-volume practices and experience with left-sided curves, which limits the generalizability of the study. The study includes two decades worth of data, and therefore could be confounded by changes and advances in surgical technique. Lastly, many providers in this group often have higher level help, and decisions on how to approach these curves may vary based on the level of support in the operating room.

## Conclusion

In AIS, there are no significant differences in surgical characteristics or outcomes between left and right-thoracic curves. Notably, curve direction does not influence correction rate, surgical time, levels fused, length of hospitalization, blood loss, pain, SRS-22 scores, or rates of reoperation, infection, or pseudoarthrosis. There are some variations in radiographic parameters postoperatively that are unlikely to be clinically significant. Regardless, these curves may require some additional thought on approach, especially if not encountered frequently in practice.

## Data Availability

The data that support the findings of this study are available from the corresponding author upon reasonable request.
